# Cell-free synthesis of human toll-like receptor 9 (TLR9): Optimization of synthesis conditions and functional analysis

**DOI:** 10.1371/journal.pone.0215897

**Published:** 2019-04-25

**Authors:** Srujan Kumar Dondapati, Georg Pietruschka, Lena Thoring, Doreen A. Wüstenhagen, Stefan Kubick

**Affiliations:** Fraunhofer Institute for Cell Therapy and Immunology (IZI), Branch Bioanalytics and Bioprocesses (IZI-BB), Potsdam, Germany; Duke University School of Medicine, UNITED STATES

## Abstract

The Toll-like receptor family belongs to the group of pathogen recognition receptors which is responsible for the discrimination of self and non-self pathogen-associated molecular patterns (PAMP’s). Toll-like receptors play an important role in the innate immunity and defects in protein expression or polymorphism is linked to various diseases such as Systemic Lupus Erythematosus (SLE). The elucidation of the underlying mechanism is crucial for future treatment and therapeutics of toll-like receptor linked diseases. Herein, we report the cell-free synthesis of human Toll-like receptor 9 (hTLR9) using CHO lysate and the continuous exchange cell-free (CECF) synthesis platform. The functionality of this protein was demonstrated by an ELISA binding assay using the ectodomain of TLR9 (TLR9-ECD).

## Introduction

The Toll-like receptor (TLR) family is an important group of pattern recognition receptors (PRRs) through which innate immunity recognizes various pathogen-associated molecular patterns (PAMPs) derived from different invasive microorganisms [1]. Toll-like receptors are type I transmembrane proteins consisting of an ectodomain, one transmembrane domain and the cytosolic Toll-interleukin 1 (IL1) receptor (TIR) domain. TLRs recognize pathogen-derived ligands by their ectodomains [2]. The structural motif of the ectodomain (ECD) contains leucine rich repeats (LRR) responsible for PAMP recognition [3]. Among the TLR family, TLR9 receptors are localized in the intracellular vesicles particularly in the endoplasmic reticulum (ER) in resting cells, and in endosomes upon stimulation by ligands [4]. TLR9 specifically recognizes single stranded DNA containing a CpG motif [5–7]. Recently, the crystal structure revealed the binding mechanism of mouse TLR9 to oligonucleotides (ODN) [8]. Before, the binding mechanism of human TLR9 (hTLR9) was proposed in an *in-silico* approach [9]. Further investigation of TLR9 is needed since targeting TLR9 receptors and modulating TLR9 signaling have emerged as important strategies as it is involved in autoimmune diseases such as systemic lupus erythematosus (SLE) [10–12]. A number of oligonucleotides modulating TLR9 immune response are in the pipeline and are in the different clinical trial phases for use in SLE treatment. Several synthetic oligonucleotide (ODN) agonists for TLR9 are currently in development for the treatment of cancer [13]. Especially, developing a faster method of TLR9 synthesis and characterization is crucial providing molecular details of the binding mechanism thereby enabling new opportunities for future drug discovery.

Cell-free protein synthesis (CFPS) represents a sophisticated alternative to the well-established cell-based expression. In CFPS, the translation machinery, retained in cell extracts after preparation, is used to produce the protein of interest. Additionally, supplements such as amino acids, RNA polymerase, salts, and energy components are provided for efficient CFPS. The advantage of CFPS over classical cell-based protein synthesis is the short synthesis time as well as the low reaction volume. Furthermore, it is an open system enabling active monitoring, rapid sampling, and direct manipulation of the protein synthesis process [14]. Thus, CFPS enables the production of difficult-to-express proteins and toxic proteins [15, 16] as well as the synthesis of modified proteins containing non-canonical amino acids [17].

CFPS can be achieved in different reaction formats, but mainly the batch format, and the continuous exchange format, based on a dialysis principle, is used for CFPS. Synthesis of proteins in a batch format is advantageous in terms of short reaction times, reproducibility, and easy handling as well as easy upscaling of reactions. Furthermore, CFPS in batch format enables parallel production of numerous proteins providing convenient and sophisticated systems for high-throughput applications [14]. The continuous exchange cell-free reaction (CECF) format is based on a dialysis principle, with a two-chamber system divided by a dialysis membrane enabling the removal of inhibitory by-products and simultaneously supplementation with substrates necessary for CFPS [18,19]. A few years ago, CFPS using CHO lysate has been successfully demonstrated in a batch mode reaction [20]. Recently, CFPS based on CHO extracts were used for the synthesis of difficult to express proteins [21]. These CHO based lysates harbor endogenous microsomal vesicles enabling translocation of membrane spanning proteins and secretory proteins. Furthermore, post-translational modifications of *de novo* synthesized membrane proteins (MPs) such as glycosylations are realized using CHO lysate [20]. CHO cell based expression is a well-established system and is approved for the large-scale synthesis of several biologics by the FDA [22]. Thus, using CHO cell lysate for cell-free protein synthesis, would enable new opportunities.

In this paper, human Toll-like receptor 9 (TLR9) is synthesized using a cell-free system based on CHO lysates. TLR9 synthesis using the CHO system is optimized towards high protein yields. This is mainly achieved by optimizing parameters such as reaction temperature, and reaction time. Subsequently, the ectodomain of TLR9 (TLR9-ECD) was synthesized for functional analysis. We have attempted to develop a simple and sensitive enzyme linked immuno assay (ELISA) based on streptavidin-biotin interactions to demonstrate the sequence, concentration and pH specific binding of TLR9-ECD to single stranded DNA containing a CpG motif.

## Material and methods

### 2.1. Template generation

Full-length human TLR9 (1–1032 amino acids) (UniProt accession: Q9NR96) with C-terminal His10 tag was purchased from GeneArt (Life Technologies) (pOA-spTLR9). Signal sequence depleted TLR9 (N-TLR9-His10) was performed in a two-step manner. Gene specific forward primer X-TLR9-F (AGAAGGAGATAAACAATGCTGGGTACCTTGCCTGCCTTCC) and reverse primer CHis-oe-TLR9-R (GTGATGGTGGTGACCCCACCAGCCACAGAGGTGATGC) were used for amplification of the TLR9 gene lacking the signal peptide sequence. NCM constructs were made by amplification of NCM sequence out of pIX3.0-NCM-Nluc using following primers: T7-F (5’-TAATACGACTCACTATAG-3’) and Mel-R (5’-GTCCGCATAGATGTAAGAAATG-3’). The TLR9 sequence was amplified using following primers: oe-Mel-TLR9-F (5’-CATTTCTTACATCTATGCGGACCTGGGTACCTTGCCTGCC-3’) and T7-R (CCCCTTGGGGCCTCTAAACGGGTCTTGAGGGGTTTTTTG). In the second step, both sequences were used and a PCR was performed using adapter primers N-0 (5’- ATGATATCTCGAGCGGCCGCTAGCTAATACGACTCACTATAGGGAGACCACAACGGTTTCCCTCTAGAAATAATTTTGTTTAACTTTAAGAAGGAGATAAACA-3’) and C-0 (5’- ATGATATCACCGGTGAATTCGGATCCAAAAAACCCCTCAAGACCCGTTTAGAGGCCCCAAGGGGTACAGATCTTGGTTAGTTAGTTA-3’) containing T7 promoter and T7 terminator as well as restriction sites for directional insertion of the obtained sequences into the linearized pIX3.0 vector. Amplified DNA templates were digested with NheI/EcoRI following over-night ligation at 16°C using T4-DNA ligase (NEB). For generation of TLR9 constructs lacking the C-terminal TIR domain (TLR9-TMD), the NCM-TLR9-His10 construct was used and the linear DNA template was amplified using N-0 (see above) and C-His-oe-TLR9-R (5’-GTGATGGTGGTGACCCCACCAGCCACAGAGGT GATGC-3’). In the second step, the DNA template was completed using X-F (5’- ATGATATCTCGAGCGGCCGCTAGCTAATACGACTCACTATAG-3’) and C-His-R (5’-ATGATATCACCGGTGAATTCGGATCCAAAAAACCCCTCAAGACCCGTTTAGAGGCCCCAAGGGGTACAGATCTTGGTTAGTTAGTTATTAATGATGGTGATGGTGGTGACCCCA-3’). A 6x His-tag was inserted into the C-terminus of the protein using a C-His primer. Finally, the TLR9 ectodomain (27–440 amino acids) was generated using again the NCM-TLR9-His10 plasmid. In the first step, the TLR9-ECD sequence was amplified using X-F (see above) and TLR9-ECD-R (5’-ATCTTG GTTAGTTAGTTATTAACAGTCCCAGGAGAGGG-3’) followed by a second amplification step using N-0 (see above) and C-0 (see above)

### 2.2. Cell-free synthesis

For eukaryotic cell-free synthesis CHO cell derived lysates were used. Lysates were prepared as described elsewhere [20]. Cell-free reactions were conducted in a coupled fashion enabling simultaneous transcription/translation of TLR9 in a volume of 50 μl. Reaction mix was composed of 40% (v/v) translationally active CHO lysate supplemented with a salt mix containing HEPES-KOH (pH 7.5, f.c. 30 mM), sodium acetate (f.c. 100 mM), magnesium acetate (f.c. 3.9 mM), potassium acetate (f.c. 150 mM), amino acids (each 100 μM), spermidine (f.c. 0.25 mM), and an energy mix composed of creatine phosphokinase (f.c. 0.1 mg/ml), creatine phosphate (f.c. 18.5 mM), ATP (1.75 mM), GTP (0.3 mM), UTP (0.3 μM), and CTP (f.c. 0.3 mM). For batch reactions, a final plasmid concentration of 60 ng/μl was used whereas for CECF reactions 120 ng/ml was used. Additionally, to prevent microbial growth, sodium azide in a final concentration of 0.02% was added. Poly[G] (30 base primer, IBA) was added in a final concentration of 4.5 μM and 20 μM for CECF and batch reactions, respectively. For transcription of TLR9 T7 RNA polymerase (Agilent) was added in a final concentration of 1 U/μl. The batch reactions were supplemented with 30 μM ^14^C-leucine (200 dpm/pmol) whereas CECF reactions were supplemented with 11 μM ^14^C-leucine (100 dpm/pmol) for further quantitative and qualitative analysis.

### 2.3. Quantitative determination of protein yield and qualitative analysis

For yield determination of radiolabeled proteins hot TCA precipitation was used. First, samples were fractionated in reaction mix, supernatant and microsomal fraction. Supernatant and microsomal fraction was obtained by centrifugation of the reaction mix at 16,000g for 15 min at 4°C. 3 μl of each fraction was transferred into a glass tube and 3 ml of 10% TCA/ 2% Caseine hydrolysate was added. The mix was boiled for 15 min in an 80°C water bath and afterwards chilled on ice for 30 min. Precipitated radiolabeled proteins were captured on a silica membrane filter (Macherey Nagel) using a vacuum filtration device (Hoefer). Dried membrane filters were placed into a scintillation tubes and 3 ml of scintillation cocktail was added and tubes were incubated for 1h under modest agitation. For measurement of the radioactivity a LS6500 Multi-Purpose scintillation counter (Beckmann Coulter) was used.

Cell-free synthesized proteins were further analyzed by SDS-PAGE and autoradiography. Therefore, 5 μl of reaction mix, supernatant, and microsomal fraction was precipitated with 45 μl MilliQ water and 150 μl ice cold acetone, respectively. Samples were incubated for at least 15 min on ice and afterwards samples were centrifuged at 16,000g for 10 min and 4°C. Acetone was removed completely and pellet was dried at 45°C for 1h in a thermomixer. Dried pellets were resuspended in 1x LDS (NuPAGE LDS sample buffer supplemented with 50 mM DTT). Afterwards samples were separated on a 10% Bis-Tris pre-cast NuPAGE SDS gel for 75 min at 150 V. SDS gels were stained using Coomassie blue solution (SimplyBlue SafeStain, Life Technologies) according to the manufacturer’s instructions. Later, SDS-gels were transferred on a Whatman paper and dried for 60 min at 70°C using a Unigeldryer 3545D, Uniequip). Autoradiography of radiolabeled proteins was performed by incubation of dried SDS-gels on a storage phosphor screen (GE Healthcare) for several days. Analysis of the screen was performed using a Typhoon TRIO+ Imager (GE Healthcare).

### 2.4. Oligodeoxynucleotides

For functional analysis of synthesized TLR9-ECD following oligodeoxynucleotides were used: ODN2006 (5’-T*C*G*T*C*G*T*T*T*T*G*T*C*G*T*T*T*T*G*T*C*G*T*T-Biotin-3’), ODN2006c (5’- T*G*C*T*G*C*T*T*T*T*G*T*G*C*T*T*T*T*G*T*G*C*T*T-Biotin-3‘). It is to mention that asterisks indicate phosphorothioate modification of the nucleic acid backbone. ODN2006 is referred as CpG sequence whereas GpC is referred as GpC/control sequence.

### 2.5. Functional analysis using ELISA

Extraction of TLR-ECD out of microsomes (referred as SN2 fraction) was performed as described elsewhere [23]. The preparation of the assay started with coating of a high binding microtiter plates (Costar) with 3 pmol streptavidin (diluted in PBS buffer) for 1h at room temperature (RT). Microtiter plates were 3x washed with wash buffer (MilliQ water/0.05% Tween). Afterwards, microtiter plates were blocked over night with 2% BSA in PBS at 4°C. After washing, plates were coated with 100 μl of biotinylated ODNs (f.c. 170 nM) for 90 min at RT. Afterwards, microtiter plates were washed 3x with wash buffer and 100 μl PBST (PBS pH 7.4/0.05% Tween) was added into each well. TLR9 samples (SN1 and SN2) were diluted in dilution buffer containing 1% BSA in PBST and samples were applied to the microtiter plates. Samples were incubated for 90 min at room temperature and afterwards, microtiter plates were washed 3x with wash buffer. The anti-TLR9 primary antibody (Abnova) was diluted 1:1000 using dilution buffer and was applied into each well of the microtiter plates. The plates were incubated over night at 4°C. After 3x washing, anti-mouse IgG-HRP secondary antibody (1:2000) (Cell signaling technology #7076, USA) was applied and incubated for 90 min at RT. Afterwards, microtiter plates were washed 3x with wash buffer and subsequently, freshly prepared self-made TMB substrate solution (5 parts 0.1 M NaH_2_PO_4_, 4 parts 0.1% H_2_O_2_/Urea, and 1 part TMB (c = 1.2 mg/ml in absolute ethanol)) was added. Microtiter plates were incubated under shaking until blue color was visible, however, incubation did not exceed 15 min. Subsequently, reaction was stopped by addition of 0.5 M H_2_SO_4_. Absorption was measured at 450 nm on a FLUOstar Omega (BMG Labtech) using Omega software. It is to mention that for the functional analysis of TLR9-ECD under acidic conditions PBS was substituted by MES. MES buffer was composed of 50 mM MES (ROTH), 150 mM NaCl (Merck), and 1 mM MgCl_2_. (Merck).

## Results and discussion

### 3.1. Optimization of cell-free hTLR9 receptor synthesis conditions

In previous work from our group, we have demonstrated that the presence of the CrPV-IRES in the corresponding vector increases the efficiency of protein synthesis in CHO based cell-free systems [21]. Recently we have reported high yield protein synthesis of EGFR and other membrane proteins using a CHO based CECF system [23,24]. In this study, we have implemented the CrPV-IRES sequence upstream of the hTLR9 gene for cell-free synthesis using a CHO based cell-free system.

#### 3.1.1. Effects of temperature and Reaction time on the CFPS

**Fig 1:**
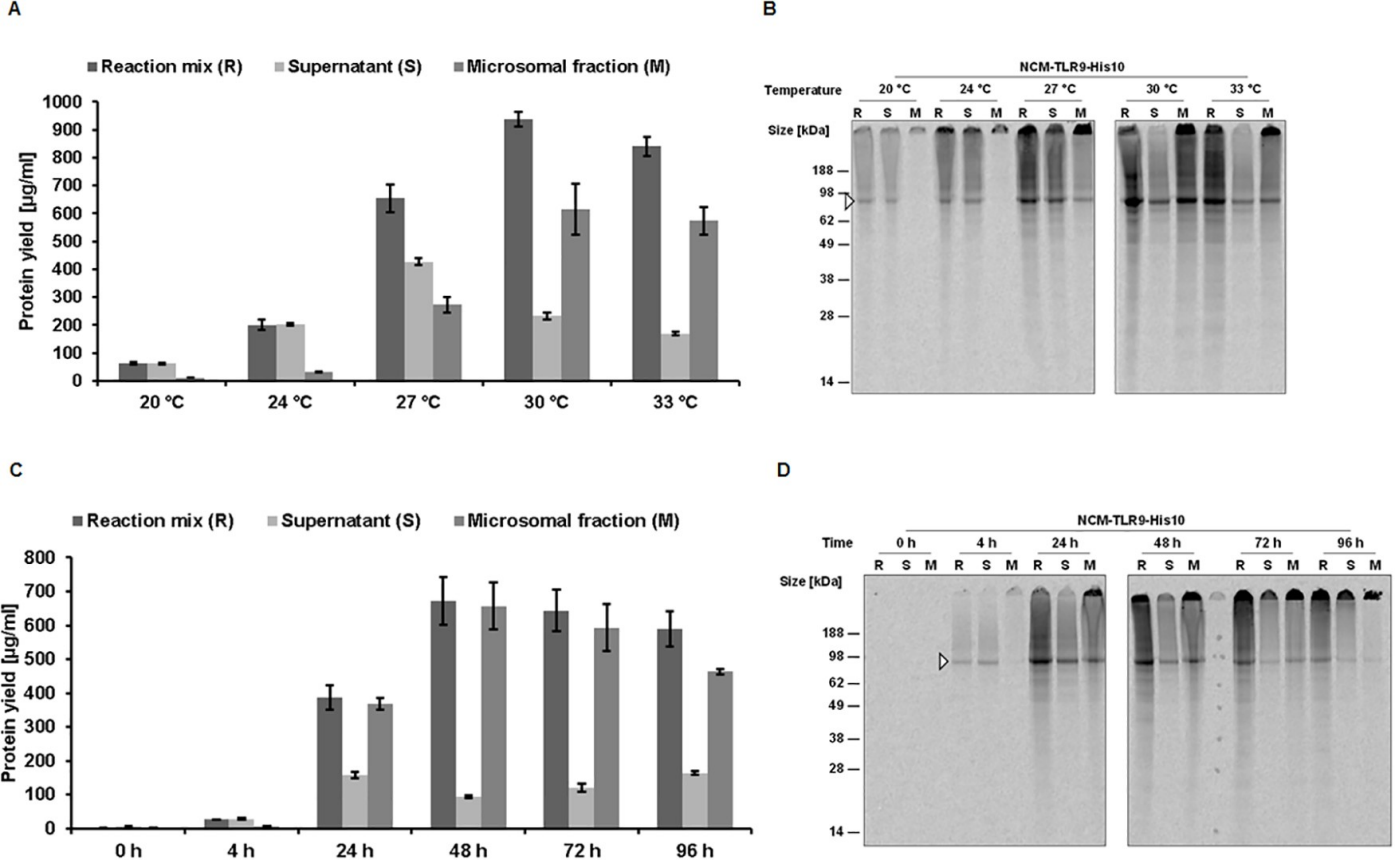
A and B) Cell-free synthesis of hTLR9 from NCM-TLR9-His10 using different reaction temperatures. Proteins were synthesized for 48h using different reaction temperatures (20°C, 24°C, 27°C, 30°C, and 33°C in the presence of radio-labelled ^14^C-leucine. C and D) Cell-free synthesis of hTLR9 using NCM-TLR9-His10 construct at different reaction times. Cell-free reaction was performed using a constant temperature of 30°C, however, reaction was stopped at different time points (0h, 4h, 24h, 48h, 72h, and 96h. A and C) Proteins were labelled with ^14^C-leucine for quantification purposes. Subsequent quantitative analysis of protein yield was performed using hot TCA precipitation and liquid scintillation counting measurement. B & D) Autoradiography of synthesized NCM-TLR9-His10 using different reaction temperatures and synthesis time points. Samples were separated on a 10% Bis-Tris pre-cast gel for 45 min at 185 V.

Our first experiments were focused on the optimization of the protein synthesis yields. The most important parameters which influence the synthesis rates in general are the reaction temperature and incubation time. The influence of these two parameters on the synthesis rates is also membrane protein specific and varies from protein to protein. Therefore, initial experiments were performed to study the influence of reaction temperature on the CFPS of NCM-hTLR9-His10. Five different temperatures within the normal temperature range (20 to 33°C) were used for the CFPS. The effect of the temperature on hTLR9 synthesis is shown in Fig 1. All protein quantification results are presented from the whole cell-free reaction mixture (R), supernatant (SN) and microsomal fractions (M) (Fig 1A and Table 1). Starting with a temperature of 20°C, the total protein yield is rather low with a determined total yield of 63 μg/ml. Increasing the temperature to 24°C improved the total protein yield drastically up to 200 μg/ml. Further rise in the temperature to 27°C increased the protein yield exponentially up to more than 3-fold (655 μg/ml) in comparison to 24°C. At 30°C, the total protein yield is around 938 μg/ml which is around 1.4 times increase in comparison to 27°C. Further increase in temperature reduces the total protein yield again by 10% (841 μg/ml). Of interest in the synthesis of membrane proteins is not only the total yield but the protein yield in the microsomal fraction (M). The amount of protein in the M fraction is important for functional studies. At 20°C and 24°C the proportion between SN/M fractions is very high (around 6) with significantly more protein present in the SN fraction (Table 1). Interestingly, this trend is changed with further increase in temperature. At 27°C the proportion is slightly decreased (1.5) compared to lower temperatures and at higher temperatures of 30°C and 33°C, the proportion between SN/M is decreased drastically (0.12 and 0.11).

**Table 1:** Overview of CECF reaction conditions and corresponding protein yields from the supernatant (SN) and microsomal (M) fractions.

Temperature	Fraction	Total Protein (μg/ml)	Time	Fraction	Total Protein (μg/ml)
**20°C**	Supernatant	61.81 +/- 2.22	**4h**	Supernatant	28.33 +/- 3.59
	Microsomal	11.27+/- 0.19		Microsomal	6.98 +/- 0.56
**24°C**	Supernatant	202.73 +/- 3.45	**24h**	Supernatant	158.51 +/- 8.76
	Microsomal	31.58 +/- 1.48		Microsomal	368.86 +/- 17.32
**27°C**	Supernatant	427.31 +/- 12.68	**48h**	Supernatant	94.10 +/- 3.23
	Microsomal	273.52 +/- 28.06		Microsomal	657.03 +/- 68.33
**30°C**	Supernatant	232.94 +/- 11.94	**72h**	Supernatant	119.29 +/- 12.04
	Microsomal	614.58 +/- 92.13		Microsomal	593.18 +/- 69.79
**33°C**	Supernatant	170.08 +/- 5.58	**96h**	Supernatant	164.11 +/- 4.55
	Microsomal	573.83 +/- 50.47		Microsomal	463.48 +/- 8.08

The qualitative analysis of the temperature dependent CFPS of hTLR9 is shown by autoradiographs (Fig 1B). At 20°C and 24°C, there is a distinct band at around 90 kDa (white triangle) visible in the R and SN fraction and absent in the M fraction. This observation resembles the SN/M proportion of the quantitation at 20°C and 24°C (Fig 1A). At 27°C, there is a distinct band in all fractions with higher band intensity in R and SN fractions and less band intensity in the M fraction. This observation also reflects the results shown in the Fig 1A. At 30°C and 33°C, the trend is reversed with a more intense band in R and M fractions compared to SN fraction which resembles the SN/M ratio of the quantitative data shown in the Fig 1A. Considering these results, including the protein yields and the SN/M proportion, we have chosen 30°C as standard temperature for further experiments.

After elucidation of the optimal synthesis temperature for the cell-free synthesis of hTLR9, we analyzed the reaction time for the CFPS of the NCM-TLR9-His10 construct. Six different incubation time points (0h, 4h, 24h, 48h, 72h and 96h) were tested for CFPS (Fig 1C). Afterwards, the optimized synthesis temperature of 30°C was used for the CFPS. After 4h of incubation, the protein yield is around 26 μg/ml. With increase in the incubation time up to 24h, the protein quantity is increased exponentially up to more than 14.9-fold with a yield of around 387 μg/ml. With further increase in time, the protein yield increased further, by 1.7-fold, to a yield of around 687 μg/ml. The protein quantity starts to decrease with further increase in the time of incubation (72h and 96h). The maximum protein yields are reached at 48h. Additionally, we have calculated the proportion of protein yields in the supernatant and microsomal fractions (SN/M ratio). At 4h, the SN/M ratio is around 4.06 with maximum protein present in the SN fraction (Table 1). At 24h, the SN/M ratio is around 0.43 with more protein in the M fraction. With further increase in the incubation time, the SN/M ratio is further decreased with the lowest ratio at 48h (0.14).

Qualitative analysis of the protein samples synthesized at different reaction times was done by SDS-PAGE followed by autoradiography (Fig 1D). At 4h, there is a single well-defined band at around 90 kDa, both, in R and SN fractions and an absence of a band in the M fraction. This is quite similar to the quantitation of the protein yield and the SN/M ratio shown in the Fig 1C. Further increase in incubation time resulted in the presence of a strong and intense band in the M fraction compared to the SN fraction. At 72h and 96h of incubation, the bands were weak in intensity at 90 kDa and most of the protein getting stuck in the wells of the stacking gel. This could be the result of aggregation of the proteins at longer time periods of incubation. Considering the quantitative and qualitative results, we have chosen 30°C and 48h as standard conditions for further experiments.

#### 3.1.2. Synthesis of hTLR9 variants by batch and CECF systems

**Fig 2:**
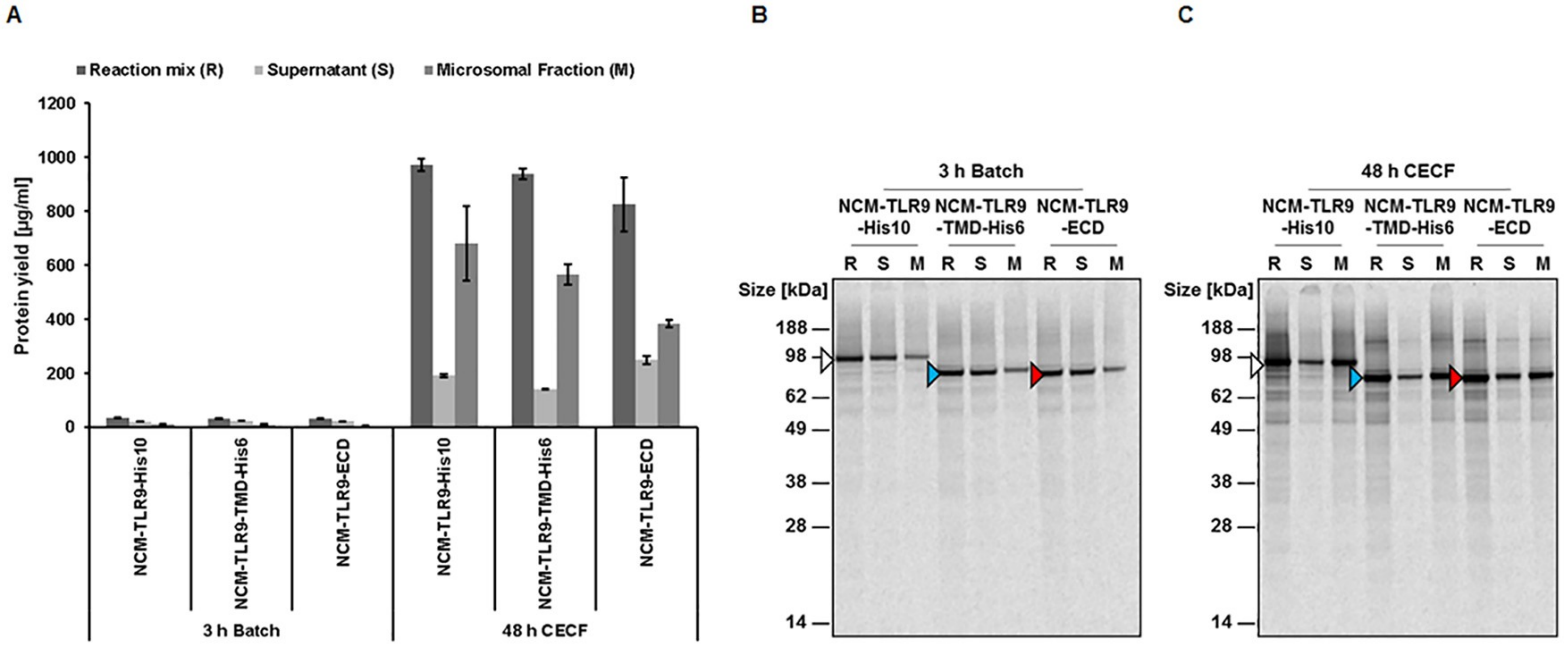
Comparison of protein yields obtained for NCM-TLR9-His10, NCM-TLR9-TMD, and NCM-TLR9-ECD using batch or CECF system. A and B) For batch mode, reaction was performed for 3h at 30°C. A and C) For CECF mode, reaction was performed using optimized conditions (see previous sections). Optimal conditions were: 48h reaction time and 30°C reaction temperature. B and C) Autoradiography of the comparison of protein yields obtained for NCM-TLR9-His10, NCM-TLR9-TMD, and NCM-TLR9-ECD using batch or CECF system, respectively. Samples were separated on a 10% Bis-Tris pre-cast gel for 75 min at 150 V. As marker, SeeBlue Plus2 Pre-Stained Protein Standard was used. Autoradiographs are modified in contrast, brightness and sharpness for better visibility.

Three different variants of hTLR9, including the TLR9-His10, NCM-TLR9-TMD, and NCM-TLR9-ECD were synthesized using the batch based and CECF systems. All reactions were performed at 30°C. For batch based system an incubation time of 3h was used. For CECF mode, the optimized conditions (30°C and 48h) were used. In the case of the batch based system, the total protein yield in the reaction mixture (R) was around 34 μg/ml, 24 μg/ml, and 22 μg/ml for TLR9-His10, TLR9-TMD-His6, and TLR9-ECD, respectively (Fig 2A and Table 2). The SN/M ratios were around 2, 2.55 and 3.37 for all three variants of proteins which shows that there is more protein present in the SN fraction compared to the M fraction (Table 2). Fig 2B shows the autoradiogram corresponding to the quantitative results obtained in the batch based system. There is a clearly defined band present at the apparent MW (MW_app_) of 90 kDa for hTLR9-his10, 75 kDa for hTLR9-TMD-His6 and hTLR9-ECD variants in the R, SN and M fractions. The intensity of the band in the autoradiogram is weak in the M fraction when compared to the R and SN fractions which reflect the SN/M ratio and quantitative data obtained with the batch based samples.

**Table 2:** Comparison of batch and CECF reaction formats for the synthesis of three variants of hTRL9.

Protein	Mode	Fraction	Time	Total Protein (μg/ml)
**TLR9-His10**	Batch	Supernatant	**3h**	21.59 +/- 0.8
		Microsomal		10.78 +/- 0.6
	CECF	Supernatant	**48h**	192.22 +/- 5.45
		Microsomal		680.13 +/- 138.17
**TLR9-TMD- His6**	Batch	Supernatant	**3h**	24.25 +/- 0.46
		Microsomal		9.51 +/- 2.53
	CECF	Supernatant	**48h**	141.36 +/- 5.61
		Microsomal		565.17 +/- 37.69
**TLR9-ECD**	Batch	Supernatant	**3h**	21.96 +/- 0.78
		Microsomal		6.52 +/- 0.34
	CECF	Supernatant	**48h**	249.25 +/- 14.69
		Microsomal		384.96 +/- 13.04

In the case of the CECF system, the protein yields are significantly increased for all protein variants. The total yield in the whole reaction mixture (R) is around 972 μg/ml for TLR9-His10, 940 μg/ml for TLR9-TMD-His6 and 826 μg/ml for TLR9-ECD (Fig 2A). The SN/M ratios for all three variants are around 0.28, 0.25 and 0.65 with most of the protein present in the microsomal fraction after centrifugation (Table 2). Qualitative analysis of all three protein variants using SDS-PAGE and subsequent autoradiography reveal clearly defined bands for all three variants at their corresponding MW_apps_. The autoradiogram reflected our quantitative observations with strong intense bands in the R and M fractions compared to this SN fraction (Fig 2B).

From the above observations, one can conclude that the CECF mode is the much superior method in terms of synthesis of high protein yields and high incorporation into the microsomes when compared to batch based system.

### 3.2. Functional characterization of hTLR9-ECD receptor using oligo binding ELISA assay

**Fig 3:**
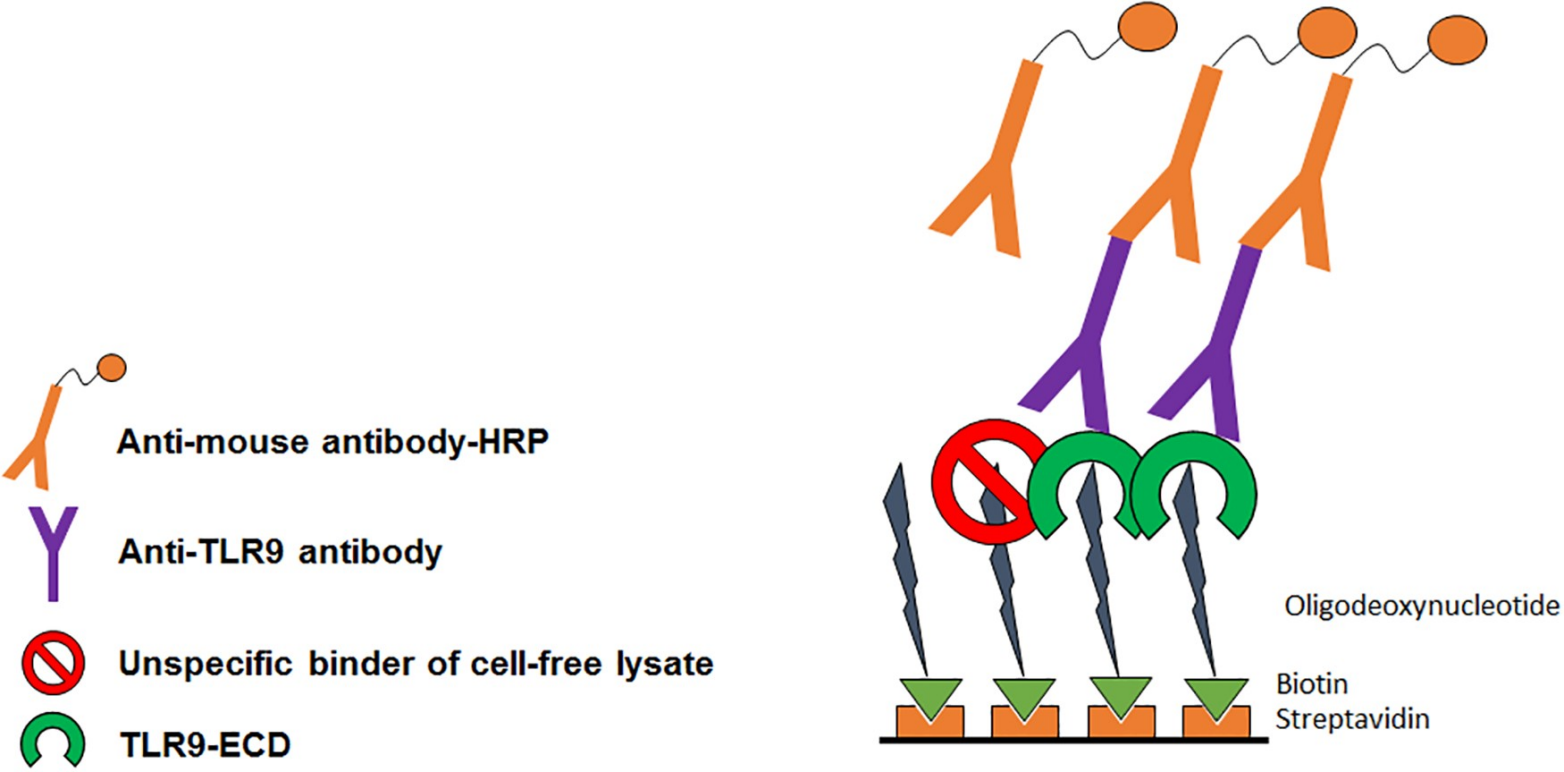
General principle of oligo based ELISA assay for analyzing the functionality of cell-free synthesized hTLR9-ECD.

After successful synthesis and characterization of the hTLR9-ECD in the CHO cell-free system, we have analyzed the functionality of the synthesized protein by a modified ELISA method where we have used an oligodeoxynucleotide sequence containing a CpG motif (ODN2006) that binds to the ECD part of hTLR9. The scheme of the oligo based ELISA assay is shown in Fig 3. First, biotinylated ODN sequences (ODN2006, ODN2006c, respectively) were immobilized on streptavidin coated microtiter plates. Further, hTLR9-ECD was added at different concentrations to the immobilized surfaces. Later, anti-TLR9 antibody was added to the bound hTLR9-ECD. Finally, detection was enabled using anti-mouse antibody conjugated with HRP. As a negative control functional analysis was done parallel with no template control samples (NTC).

**Fig 4:**
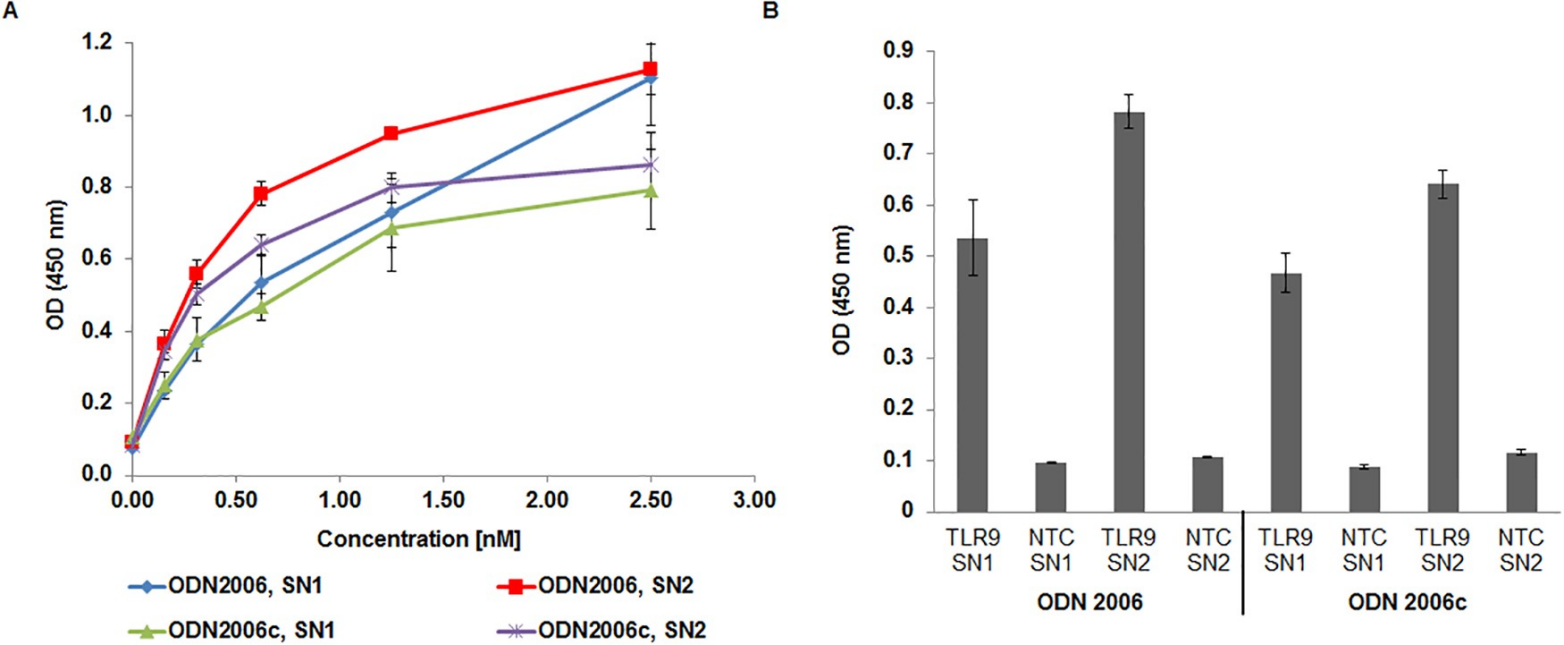
Functional analysis of cell-free synthesized TLR9-ECD by ELISA. A) Binding curves of TLR9-ECD supernatant fraction 1 (SN1) and supernatant 2 (SN2) are shown. ELISA was performed at pH 7.4. B) Absorbance values resulted from the binding of 0.64nM hTLR9 and NTC to ODNs plotted for all the samples. SN1 fraction was obtained after centrifugation of reaction mix for 15 min at 16,000g and 4°C. SN2 fraction was obtained after incubation of microsomes with PBS + 0.2% DDM and subsequent centrifugation for 15 min at 16,000g and 4°C. Biotinylated streptavidin was used as control (S1 Fig)

Initial oligo based ELISA experiments were performed at pH 7.4. The binding potential of hTLR9-ECD on ODN2006 and ODN2006c is shown in the Fig 4. Both the SN1 fractions as well as the SN2 fractions were used to analyze the interactions. A concentration-response binding curve is plotted to test the sensitivity of the protein-oligonucleotide interactions. For the SN2 fraction, a linear response is visible up to 0.64nM protein and the response curve started to bend towards the concentration axis at higher concentrations. At 2.5nM, the binding capacity of the SN2 fraction towards ODN2006 is higher compared to ODN2006c. Further, no binding is observed for any of the no template control (NTC) sample. The binding profile for the SN1 fraction is quite similar; however, the binding affinity is lower. In the case of ODN2006, the SN1 fraction did not show any saturation at 2.5 nM. The NTC samples do not show any binding which clearly shows that the binding related absorbance is more due to the specific interaction between ODN and ECD and not due to unspecific binding. Moreover, with increase in the concentration, the gap between the absorbance values due to SN-ODN binding increases. Therefore, we have plotted the absorbance values of all our experiments at 0.64 nM where we notice an increase in the specificity of the SN2 binding to the ODNs (Fig 4A). From Fig 4B, we can notice that TLR9-SN2 binding to ODN2006 shows a higher absorbance compared to binding to ODN 2006c. All control experiments show comparatively a very low response. This demonstrates the specificity of the hTLR9-ECD in binding to CpG containing oligodeoxynucleotides. The limit of detection (LOD) for all the samples is around 0.16 nM. The maximum absorbance of TLR9 SN1 is around 0.8 at a concentration of 2.5 nM (SN1-2006c). The sensitivity calculated from the logarithmic plot for achieving the same OD450 nm is around 0.72 nM, 1.46 nM and 1.22 nM for SN2-2006, SN2-2006c and SN1-2006 interactions.

**Fig 5:**
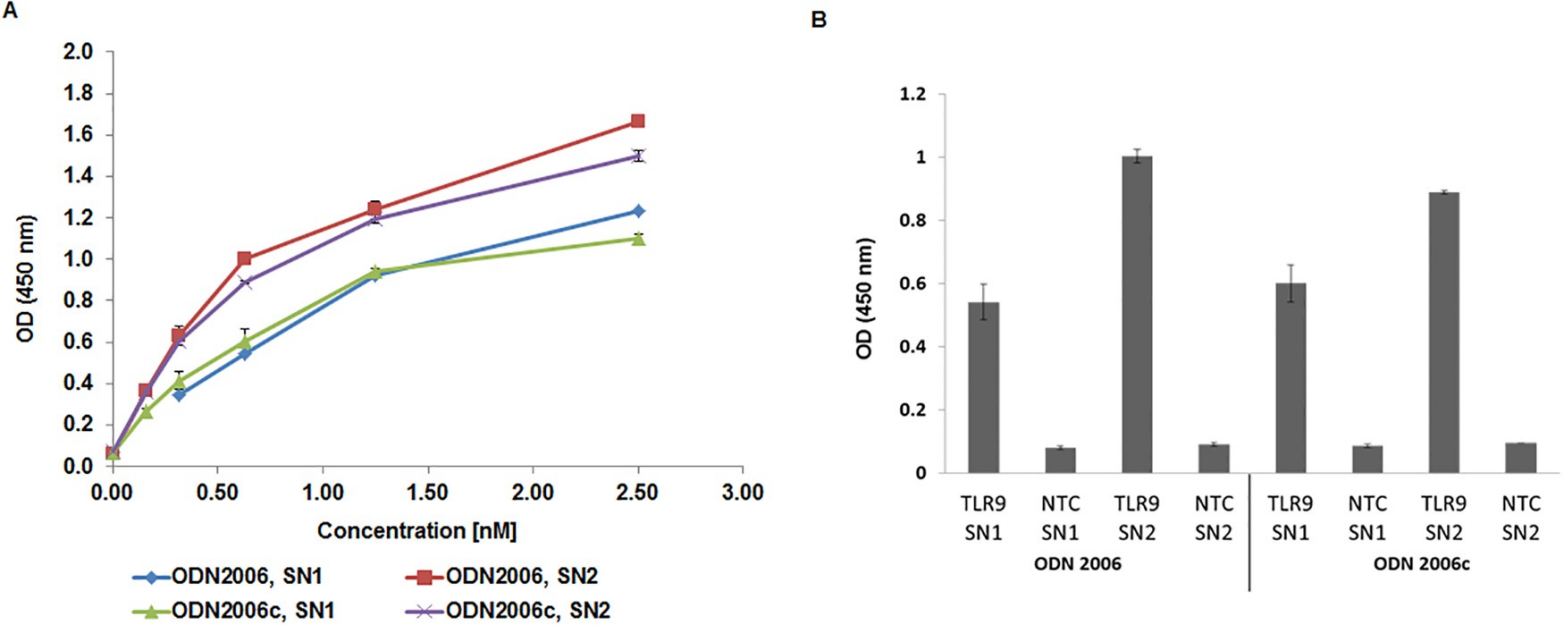
Functional analysis of cell-free synthesized TLR9-ECD by ELISA. A) Binding curves of TLR9-ECD supernatant fraction 1 (SN1) and supernatant 2 (SN2) are shown. ELISA was performed at pH 6.5. B) Absorbance values resulted from the binding of 0.64 nM hTLR9 and NTC to ODNs plotted for all the samples SN1 fraction was obtained after centrifugation of reaction mix for 15 min at 16,000g and 4°C. SN2 fraction was obtained after incubation of microsomes with PBS + 0.2% DDM and subsequent centrifugation for 15 min at 16,000g and 4°C.

**Fig 6:**
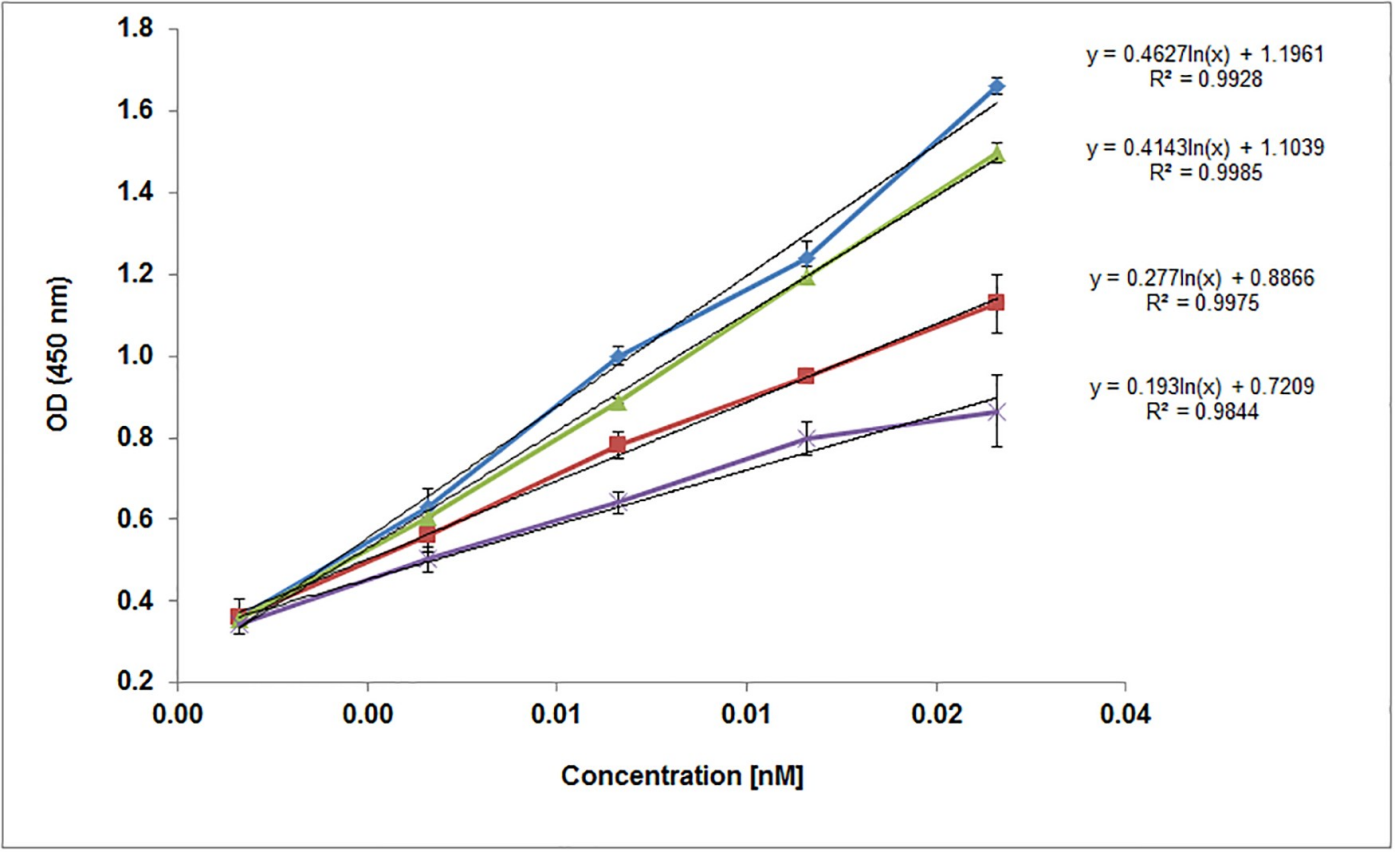
Variation of absorption at 450nm for different concentrations obtained from the binding of SN2 fractions of TLR9-ECD on ODN2006 (Blue for pH 6.5 and Red for pH 7.4) and ODN2006c (Green for pH 6.5 and Violet for pH 7.4). Results from Figs 4 and 5 were used and combined to render this illustration.

It was demonstrated that TLR9 binds to single-stranded CpG ODNs in a sequence and pH dependent manner [14]. Our next experiments were focused on the investigation of the binding response at pH 6.5. Fig 5A shows the concentration dependent response curve for both SN1 and SN2 fractions. The binding trend is similar as observed with pH 7.4 but the response is slightly higher for all concentrations. The SN2 fractions shows higher affinity to ODNs compared to SN1 fractions. Response with ODNs is linear until 0.64nM concentration and thereafter the curve starts to show bend towards Y axis at higher concentrations. All the negative controls show comparatively negligible response. In comparison to pH 7.4, SN2 fractions show a significantly higher affinity to ODNs compared to SN1 fractions. There is a very low background signal from the negative control samples which indicate that the binding related absorbance is more due to the specific affinity between ODN and ECD and not due to unspecific binding. Absorbance values due to binding for all the experiments at a concentration of 0.64 nM are shown in the Fig 5B. There is noticeable difference between the absorbance values due to binding to ODN2006 and ODN2006c for the SN2 fraction. All control experiments showed comparatively low responses. This demonstrates the specificity of hTLR9-ECD in binding to ODN 2006 samples.. The limit of detection (LOD) for all the samples is around 0.16 nM. The maximum absorbance of TLR9 SN1 is around 1.10 at a concentration of 2.5 nM (SN1-2006c). The sensitivity calculated from the logarithmic plot (equation displayed in Fig 6) for achieving the same OD450 nm is around 0.82 nM, 1.0 nM and 1.76 nM for SN2-2006, SN2-2006c and SN1-2006 interactions.

Finally, the logarithmic concentration dependent absorbance values due to the binding of SN2 fractions with ODNs at different pHs are shown in Fig 6.

Fig 6 illustrates the variation of absorbance with increase in the concentration of SN1 and SN2 concentrations at pH 6.5 and pH 7.4 respectively. There is a higher affinity at pH 6.5 compared to pH 7.4. But the difference in the binding affinity between ODN2006 and ODN 2006c was higher with pH 7.4 compared to pH 6.5 (S2 Fig). In conclusion, the difference in binding affinity of TLR9-ECD to oligodeoxynucleotides used in this study at any pH is only observed starting from a concentration of 0.31 nM. Additionally, with increase in the concentration of TLR9, the binding affinity increased. There is a constant variation in the absorbance with change in the protein concentration at both pHs with a good correlation between logarithmic concentration of TLR9 and OD450 nm values (R2>0.99 for all the samples).

## Discussion

Herein, we present for the first time the synthesis of hTLR9 in a cell-free system using CHO lysates. With this synthesis platform it was possible to obtain protein yields of hTLR9 with up to 680 μg/ml in the microsomal fraction (Fig 2A, Table 2). CHO cell lysate was usually used for fundamental understanding of mRNA translation [25,26]. Furthermore, Broedel *et al*. demonstrated in 2013 for the first time the cell-free synthesis of heparin-binding EGF-like growth factor receptor, epidermal growth factor receptor, and erythropoietin using the CHO system [27]. By fusion of an internal ribosomal entry site (IRES) of the Cricket Paralysis Virus (CrPV), it was possible to synthesize proteins in a cell-free dependent manner using a mammalian cell lysate. It must be emphasized that for optimal high yield protein synthesis it was also necessary to substitute the normally used AUG start codon by a GCU codon [28]. By this substitution, an increase of luciferase synthesis could be achieved in a batch based system using the CHO lysate [20,27]. Further, the synthesis has been performed in a coupled system, enabling simultaneous transcription and translation, to save time and labor compared to the linked system [29]. Using the coupled system has also the advantage that it can be implemented in a variety of other cell-free platforms [23,30,31]. Furthermore, we used the continuous exchange cell-free (CECF) synthesis technology for high yield protein production. The advantages of CECF in CFPS were extensively discussed by Thoring *et al* [21,24]. Prior to high yield protein synthesis, several optimizations are mandatory. It was shown that the DNA template has an influence on protein yield. Using linear DNA templates resulted in a lower protein yield compared to a circular DNA template [21]. Further optimization could be achieved by adjusting the T7 polymerase concentration in the synthesis reaction as well as the addition of molecular crowding reagents like PEG [21]. Additionally, the optimization of the magnesium concentration has a significant impact in CFPS. By adjustment of the magnesium concentration from 3.9mM used for batch reactions to 22.5 mM, a 3-fold increase in protein yield could be obtained [24]. In this work, we focused mainly on the optimization of the reaction time and reaction temperature. We could show that a prolonged reaction time is linked to an increase in protein yield as demonstrated for the full-length construct NCM-TLR9-His10 (Fig 1C). However, the maximum yield was achieved after 48h and further increase in reaction time did not result in a higher protein yield. Next, the elucidation of the proper reaction temperature was another crucial parameter for high yield CFPS (Fig 1A).

The advantage of CECF over the batch system was herein demonstrated. In general, the synthesis of a protein using the batch system is completed after several hours due to the depletion of energy components and the accumulation of inhibitory by-products. In contrast, the synthesis of a protein using CECF can be extended up to 48h with steady increase in the overall protein yield (Fig 1C). These results resemble the results obtained from other studies [21,24]. Although higher yields are expected in the case of CECF for 48h compared to shorter reaction times, direct comparison with a different format of cell-free system (batch based) demonstrated the advantage of using CECF system, both, in terms of quantity and SN/M ratio for hTLR9. Depending on the type of protein and its application the synthesis method can be chosen. In our study, 2h batch based system in general is sufficient for analyzing the functionality. But, for preparing several dilutions and to perform multidimensional titrations CECF is an ideal choice.

Recently, a comparison of cell-based expression systems with the cell-free system using CHO lysate revealed that the cell-free based system was superior to CHO and HEK cell expression systems. The cell-free synthesized hBMP2 had a 20-fold higher yield compared to the cell-based system [32]. Additionally, the overall productivity of the cell-free system was much higher compared to the cell-based system. This emphasizes the great opportunity of the cell-free systems.

In this study, the functional binding of hTLR9-ECD to CpG oligodeoxynucleotides ODN2006 and the control sequence ODN2006c was demonstrated (Figs 4 and 5). This binding is sequence and pH dependent which is consistent with the results of mTLR9-ECD-Fc and hTLR9-ECD-Fc observed by Rutz *et al* [33]. Furthermore, a sequence and pH dependent binding of horse TLR9 (EcTLR9) was demonstrated using isothermal titration calorimetry (ITC) [8]. The K_d_ values of EcTLR9 varied up to 20-fold depending on the pH. Binding affinities are around 0.63nM in our study and are comparatively higher than the values reported in the literature [34, 35]. But the difference between the binding affinity in recognizing CPG and non-CPG is relatively smaller in our study when compared to the 62nM (CPG) and 153 (non-CPG) derivatives reported in the literature [35]. This could be due to the fact that our assay is performed with a non-purified TLR9 sample and additionally the binding affinities were estimated using different types of detection system.

Additionally, binding was not only sequence dependent but also independent of proteolytical processing. Proteolytical processing seems to be a common motif for nucleic acid sensing receptors since TLR3 and TLR7 are also regulated by proteolytical processing [36–38]. In general, binding of nucleic acid sensing toll-like receptors is enabled through the horse-shoe shaped ectodomain [8, 39]. To achieve full functionality of the receptor, the ectodomain is processed at the Z-loop performed by endolysosomal proteases like cathepsins and asparagine endopeptidase [36,37,40,41]. In case of TLR9, this proteolytical processing enables dimerization of the receptor with subsequent activation and downstream signaling.

Toll-like receptors play an essential role in the innate immune system. They are involved in the recognition of self and non-self pathogen-associated molecular patterns (PAMP’s). An aberrant modulation in the function of toll-like receptors is responsible for certain autoimmune diseases and cancer [10–13]. Therefore, the need for new drugs is of outstanding importance. Cell-free synthesis enables the possibility of studying biophysical interactions of the Toll-like receptor with its ligands. This is important for the development of future therapeutics. Compared to other assays available for TLR9 detection, our assay can be automated and can be performed parallel with small reagent volumes on multiple microtiter plates. This is advantage for high-throughput identification of compounds interacting with TLR9. With this we could demonstrate the cell-free synthesis and fast generation of mutant versions of a functional TLR9 protein which is of vital importance in the innate immune system.

## Supporting information

S1 Fig Binding of TLR9-ECD SN2 and NTC SN2 fractions on ODN2006 and ODN2006c at pH 7.4. (TIF)

S2 Fig Binding of TLR9-ECD SN2 fraction on ODN2006 and ODN2006c at pH 7.4. (TIF)
