# Supplementary figures and images for: Cell-free synthesis of human toll-like receptor 9 (TLR9): Optimization of synthesis conditions and functional analysis
Source: PLoS One. 2019 Apr 25;14(4):e0215897. doi: 10.1371/journal.pone.0215897 (PMC6483205; doi:10.1371/journal.pone.0215897)

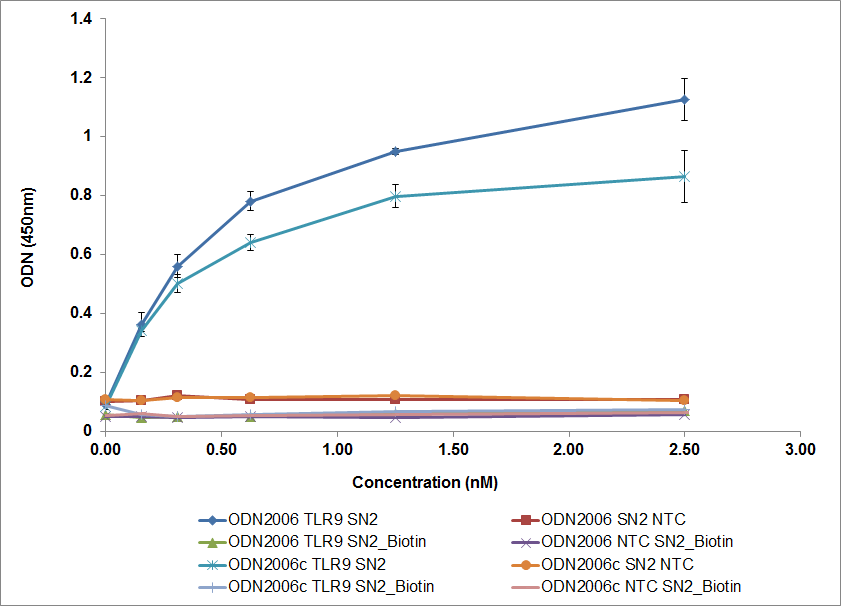

Supplement: S1 Fig — (TIF) [file pone.0215897.s001.tif]

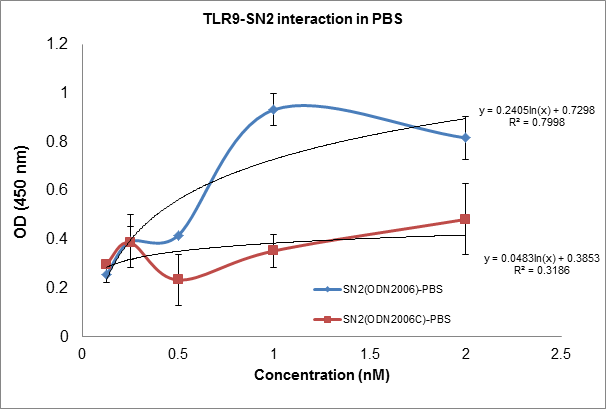

Supplement: S2 Fig — (TIF) [file pone.0215897.s002.tif]
